# In vitro metabolism and metabolic effects of ajulemic acid, a synthetic cannabinoid agonist

**DOI:** 10.1002/prp2.17

**Published:** 2013-12-15

**Authors:** Sumner H Burstein, Mark A Tepper

**Affiliations:** JB Therapeutics, Inc.508 Dudley Road, Suite 100, Newton, Massachusetts, 02459

**Keywords:** Ajulemic acid, cannabinoid, metabolism

## Abstract

Ajulemic acid is a synthetic analog of Δ^8^-THC-11-oic acid, the terminal metabolite of Δ^8^-THC. Unlike Δ^9^-THC, the psychoactive principle of *Cannabis,* it shows potent anti-inflammatory action and has minimal CNS cannabimimetic activity. Its in vitro metabolism by hepatocytes from rats, dogs, cynomolgus monkeys and humans was studied and the results are reported here. Five metabolites, M1 to M5, were observed in human hepatocyte incubations. One metabolite, M5, a glucuronide, was observed in the chromatogram of canine hepatocyte incubations. In monkey hepatocyte incubations, M5 was observed in the chromatograms of both the 120 and 240 min samples, trace metabolite M1 (side-chain hydroxyl) was observed in the 120 min samples, and trace metabolite M4 (side-chain dehydrogenation) was observed in the 240 min samples. No metabolites were found in the rat hepatocyte incubations. Unchanged amounts of ajulemic acid detected after the 2-h incubation were 103%, 90%, 86%, and 83% for rat, dog, monkey, and human hepatocytes, respectively. Additional studies were done to ascertain if ajulemic acid can inhibit the activities of five principal human cytochrome P450 isozymes; CYP1A2, CYP2C9, CYP2C19, CYP2D6, and CYP3A4/5. In contrast to the phytocannabinoids Δ^9^-THC and CBD, no significant inhibition of cytochrome activity was observed. These data further support the conclusions reached in earlier reports on ajulemic acid's high margin of safety and suggest that it undergoes minimal metabolism and is not likely to interfere with the normal metabolism of drugs or endogenous substances.

## Introduction

Researchers have long sought potent synthetic analogs of the active ingredients of *Cannabis sativa*. Their goal has been to develop clinically useful drugs, especially for the treatment of pain and inflammation that are free of the psychotropic effects associated with recreational use of *Cannabis*. Ajulemic acid (AJA) is a potential candidate that in preclinical studies displayed similar the properties to the non-steroidal anti-inflammatory drugs (NSAIDs), but without their undesirable side effects, for example, ulcerogenicity (Burstein 2002, 2005; Zurier 2003; Wiley 2005).

Initial trials in healthy human subjects, as well as in patients with chronic neuropathic pain, demonstrated an absence of psychotropic actions (Karst et al. 2003; Karst 2007). Moreover, it proved to be more effective than placebo in reducing pain as measured by the visual analog scale. Unlike the narcotic analgesics, signs of dependency were not observed after withdrawal of the drug at the end of the 1-week treatment period. The potential effectiveness and safety of AJA (20 mg and 40 mg b.i.d. for 1 week) has been demonstrated in 21 patients with refractory neuropathic pain in a single-center crossover study (Karst et al. 2003). In addition, results from an earlier phase 1 study demonstrated approximately linear pharmacokinetics in cohorts of eight subjects per dose (two placebo/six active), given doses of 1, 3, 5, and 10 mg of AJA, respectively (Atlantic Pharma, New York, NY).

In one of its actions, it favorably influences the balance of pro- and anti-inflammatory eicosanoids, in particular prostaglandin J_2_ (Stebulis et al. 2008) and lipoxin A_4_ (Zurier et al. 2009). This mechanism of action requires further pharmacological study to be fully elucidated. In vivo studies have demonstrated its anti-hyperalgesic, anti-allodynic and anti-inflammatory potential (Zurier 2003), which were confirmed in a clinical phase 2a proof of concept study in refractory, severe, long-standing neuropathic pain of traumatic origin (Karst et al. 2003). Nonclinical and early clinical studies have established a good safety and tolerability profile. Clinical and nonclinical data constitute a consistent dataset supporting the efficacy and safety of AJA. It is central nervous system-excluded to some degree (Dyson et al. 2005) and is devoid of psychoactivity. It is currently being studied for possible use in the treatment of fibrotic diseases, such as scleroderma.

An understanding of the metabolism of AJA is important for an understanding of its pharmacology and mechanism of action. Active metabolites have the potential to bind to other receptors and have actions not in common with AJA that would affect its pharmacodynamic properties in vivo. Moreover, species-specific toxic metabolites may occur such as those reported for Nabilone, a closely related synthetic cannabinoid analog (Hanasono et al. 1987) where a unique canine metabolite showed significant toxicity. Harvey and Brown (1991), in a comprehensive study of cannabinoid metabolism, described species specific hydroxylation patterns for the phytocannabinoids. The entire subject of cannabinoid metabolism has been reviewed by Huestis (2007) who described the extensive metabolic conversions of these molecules. She describes phase-I oxidation reactions of tetrahydrocannabinol (THC) that include allylic and aliphatic hydroxylations, oxidation of alcohols to ketones and acids, *β*-oxidation, and degradation of the pentyl side chain. Conjugation with glucuronic acid is a common phase-II reaction. This is in sharp contrast to AJA where only minimal biotransformations occur both in vitro and in vivo (vide infra).

The objective of the study reported here was to investigate the in vitro biotransformation of AJA in cryopreserved hepatocytes from Sprague-Dawley rats, beagle dogs, cynomolgus monkeys, and humans. Metabolite profiles of AJA after incubation with hepatocytes from these four species were compared with metabolites of AJA, characterized using HPLC/MS and HPLC/Tandem MS techniques. A further objective of this study was to evaluate the inhibitory potential of AJA on five principal human cytochrome P450 isozymes. CYP1A2, CYP2C9, CYP2C19, CYP2D6, and CYP3A4/5 activities were determined using phenacetin, tolbutamide, S-mephenytoin, dextromethorphan, midazolam, and testosterone, respectively, as the probe substrates in pooled human hepatic microsomes. CYP3A4 is the most abundant P450 form in human liver microsomes and accounts for about 28% of total CYP in human liver. CYP3A5 is a closely related isozyme of CYP3A4, and accounts for 0–8% of total CYP in human liver. Midazolam and testosterone are substrates for both CYP3A4 and CYP3A5.

## Materials and Methods

### Cells

Pooled rat (Sprague Dawley, male and female), canine (Beagle, male and female), and human (male and female) cryopreserved hepatocytes were obtained from XenoTech, L.L.C. (Lenexa, KS). Monkey (Cynomolgus, male and female) cryopreserved hepatocytes were obtained from Cellz Direct, Inc. (Durham, NC). Both female and male hepatocytes were pooled and centrifuged through a percoll preparation (Hepatocytes Isolation Kit; Xenotech LLC, Lenexa, KS). Viability was assessed using trypan blue exclusion, which was included in the hepatocyte isolation kit. After washing, the cells were diluted to 1 × 10^6^ cells/mL in Krebs-Henseleit Buffer (pH 7.4) supplemented with 1 mmol/L sodium pyruvate and 5 mmol/L glucose.

### Ajulemic acid

The synthesis of AJA can be found in Data S1.

### Metabolism study design

Hepatocyte incubation samples were collected in triplicate after incubation of 10 μmol/L AJA with cryopreserved rat, dog, monkey, and human hepatocytes. At the end of the incubation, hepatocyte mixtures were quenched with acetonitrile and stored frozen at −20°C. On the day of analysis, frozen samples were thawed out, vortexed, and centrifuged at 15,000 rpm for 10 min. Supernatants were transferred to auto-sampler injection vials for LC/MS analysis. For rat, canine, monkey, and human hepatocyte incubations, a 100 μmol/L stock solution of AJA was prepared in dimethyl sulfoxide (DMSO). Both positive controls, 7-hydroxycoumarin and 7-ethoxycoumarin, were also prepared at 100 μmol/L in DMSO. The positive control 7-ethoxycoumarin was evaluated for the cryopreserved hepatocytes of all four species.

Incubations of 10 μmol/L AJA with rat, canine, monkey, and human hepatocytes was carried out in triplicate. Before each incubation, rat, canine, monkey or human cryopreserved hepatocytes were thawed out following *Xenotech Protocol for Thawing Cryopreserved Hepatocytes* for preparation of hepatocyte suspensions. After the desired cell concentration of 1 × 10^6^ was reached by dilution, cells in suspension buffer (Kreb-Henseleit Buffer, pH 7.4) were added into glass tubes, and the reaction was initiated by adding AJA (10 μmol/L final incubation concentration) into the incubation mixture. Total incubation volume was 0.5 mL and the incubation time periods were 0, 2, and 4 h for each species. Vehicle controls without cells were included in the incubations in duplicate, and the sampling times were at 0, 2, and 4 h. At the end of each time period, 500 μL acetonitrile was added to the reaction tube to terminate any metabolic reaction. All incubation samples were snap frozen on dry ice. Reaction mixtures were stored at −20°C until LC/MS/MS analysis was performed (Table 1).

**Table 1:** HPLC/MS conditions

HPLC	
Column type	Synergy 4u MAX-RP 80Å, 4.6 × 250 mm
Mobile phases	A: 0.1% NH_4_OH/high purity water
	B: 0.1% NH_4_OH/acetonitrile
Gradient program	1. 0 min: 75% A, 25% B
	2. 15 min: 55% A, 45% B
	3. 25 min: 10% A, 90% B
	4. 30 min: 10% A, 90% B
	5. 31 min: 75% A, 25% B
	6. 39 min: 75% A, 25% B
Flow rate	800 μL/min
Analysis time	25 min
Injection volume	200 μL
Mass spectrometry	
Sheath gas	40
Ion spray voltage	2 kV
Capillary temperature	300°C
Capillary voltage	−38 V
Tube lens	−105 V
Ionization mode	Negative ESI

The system for metabolite profiling and identification consisted of a CTC LEAP HTC autosampler, a Surveyor HPLC pump, and an LTQ mass spectrometer. The HPLC/MS system was controlled by Xcalibur software. The following are the conditions for HPLC and mass spectrometry.

HPLC/tandem MS was used for metabolite profiling and identification. Metabolites of AJA were separated using reverse phase chromatography, and detected by ESI mass spectrometry. Total ion chromatograms (TIC) of incubation samples of AJA with rat, canine, monkey, and human hepatocytes were obtained. Putative metabolites of AJA were searched for using the extracted ion chromatograms of their molecular ions. Tandem MS of these molecular ions was performed, and the structures of the metabolites were proposed by interpretation of their mass spectra.

Positive controls, 7-ethoxycoumarin and 7-hydroxycoumarin at 1 μmol/L, were incubated concurrently with the test article, in duplicate, in rat, canine, monkey, and human hepatocytes. The incubation conditions are described above. The sampling times were at 0, 15, 30, and 120 min for 7-ethoxycoumarin, and 0, 30, and 120 min for 7-hydroxycoumarin. Total incubation volume was 0.3 mL. The metabolic reactions were stopped at the end of each time period by addition of 300 μL of acetonitrile containing carbutamide at 1 μg/mL as an internal standard. The quenched samples were centrifuged at 3000 rpm for 10 min, and the supernatant was transferred into a clean tube and stored at –20°C until the LC/MS/MS analysis.

### Design for inhibition studies

#### Microsome preparation

Adapted from a previous report (Dierks et al. 2001). Human liver microsomes were prepared by differential ultracentrifugation from donor human liver samples obtained from IIAM (Scranton, PA) according to published protocols (Lake et al. 1987). Microsomal cytochrome P450 content was determined by CO-difference spectrum (Omura and Sato, 1964). Protein content was determined by the Bradford method (Bradford, 1976; Macart and Gerbaut, 1982). Microsomes used in these studies were pooled from a minimum of five individuals.

The AJA primary stock solution was prepared in DMSO at 20 mmol/L; the working solution was prepared in 0.1 mol/L potassium phosphate buffer (pH 7.4) containing 2% ACN and 0.4% DMSO. From the 200 μmol/L working solution, 1:3 serial dilutions were made to generate ten concentrations of AJA. The dilutions were made in 0.1 mol/L potassium phosphate buffer containing 2% ACN. Ten final concentrations of AJA were evaluated in triplicate (0, 0.0076, 0.022, 0.069, 0.21, 0.62, 1.85, 5.55, 16.7, and 50 μmol/L). Positive control inhibitors for each isoform was analyzed along with test compound and compared to literature values to confirm assay reliability. Stock solutions of each of the following positive controls were prepared in DMSO and stored at –20°C until use.

1. Furafylline (CYP1A2) highest final concentration 100 μmol/L
2. Sulfaphenazole (CYP2C9) highest final concentration 10 μmol/L
3. Omeprazole (CYP2C19) highest final concentration 100 μmol/L
4. Quinidine (CYP2D6) highest final concentration 10 μmol/L
5. Ketoconazole (CYP3A4/5) highest final concentration 10 μmol/L

In a 96-well plate, the different concentrations of AJA and the positive control inhibitors were added to specified wells. Microsomes were thawed and diluted with 100 mmol/L potassium phosphate buffer (pH 7.4). Microsomal protein concentration in the final reaction mixture was 0.5 mg/mL. The appropriate substrate was added to the CYP specific reactions. To initiate the reaction, NADPH (2 mmol/L) containing 3 mmol/L MgCl_2_ was added to each well. Furafylline was pre-incubated for 5 min at 37°C with AJA, 2 mmol/L NADPH and microsomes before the addition of the substrate. All other isoforms were pre-incubated for 5 min at 37°C with AJA, substrate, and microsomes before the addition of 2 mmol/L NADPH. The total incubation volume was 100 μL. After the designated incubation time for each reaction, one volume of ice-cold acetonitrile was added to each well to terminate the reaction. Plates were left on ice or in a refrigerator for 30 min. The plates were centrifuged for ∼5 min at 2000 rpm to pellet the microsomal protein. After centrifugation, aliquots of supernatant were pooled and transferred into a fresh 96-well plate for LC/MS/MS analysis. An aliquot (130 μL) of supernatant at each concentration level of CYP2C9 and CYP2C19 assays and 50 μL of supernatant at each concentration level of CYP1A2, CYP2D6, and CYP3A4/5 assays were pooled for analysis.

Metabolites, acetaminophen for CYP1A2, 4-hydroxytolbutamide for CYP2C9, 4′-hydroxymephenytoin for CYP2C19, dextrorphan for CYP2D6, 6ß-hydroxytestosterone, and 1′-hydroxymidazolam for CYP3A4/5, in pooled samples were analyzed using electrospray ionization (ESI) techniques in the positive ion mode. An internal standard antipyrine (2 μmol/L) was used in the sample analysis. Calibration standard curves of acetaminophen, 4-hydroxytolbutamide, 4′-hydroxymephenytoin, dextrorphan, 6ß-hydroxytestosterone, 1′-hydroxymidazolam, were prepared in control human liver microsomes. Quality control (QC) samples (3 or 4 levels) were included in the analytical run to ensure assay performance.

### Data analysis

Percent of vehicle control was calculated using the Excel® software program (version 97, Microsoft Corporation). The pmol/min/mg values determined by LC/MS/MS analysis were used for this calculation. The pmol/min/mg microsomal protein values at each concentration of test article were compared to the pmol/min/mg value at the highest concentration of test article (vehicle control) to give the percent of vehicle control. The IC-50 of positive controls and AJA was determined using Win Nonlin software (Pharsight Corp.). The IC-50 values for positive controls analyzed in this experiment were compared with the reported literature values (Dierks et al. 2001).

## Results

**Figure 1:**
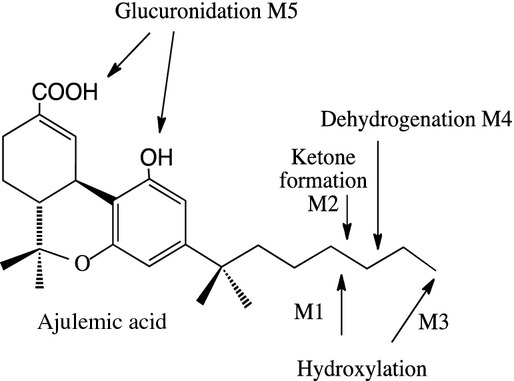
Proposed pathways for hepatocyte metabolism of AJA. The positions of the double bond in M4, the keto group in M2 and the hydroxyl group in M1 are not known. The location of the glucuronidation (M5) could not be determined.

**Figure 2:**
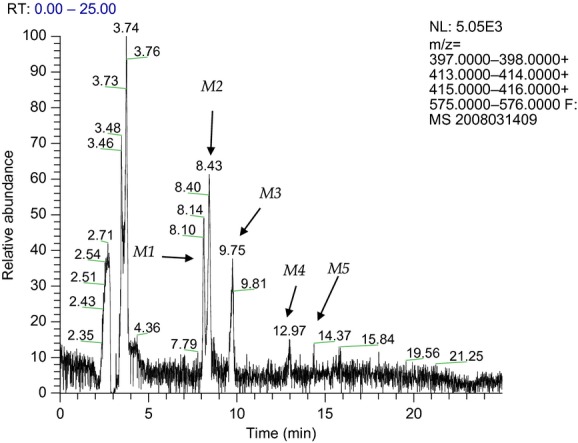
Extracted ion chromatogram of AJA metabolites from human hepatocyte incubation samples at 120 min incubation times. Conditions as described in Table 1.

AJA was analyzed using HPLC Tandem MS, and its retention time was 16.14 min on the system described in Table 1. The product ion spectrum of *m/z* 399.5 showed a base peak at *m/z* 355 and a major peak at *m/z* 381; the chromatogram and the proposed structures of the metabolites are shown in Figures 1 and 2, and Table 2. The metabolite profile of AJA after incubation with human hepatocytes showed more metabolites than the other three species. The incubations of AJA with canine hepatocytes showed one metabolite, M5. The incubations of AJA with monkey hepatocytes also showed metabolite, M5, and two trace metabolites, M1 and M4. The incubations of AJA with rat hepatocytes did not show any observable metabolites.

**Table 2:** Relative HPLC peak intensities of AJA and its metabolites from rat, canine, monkey, and human hepatocyte incubation samples

	Metabolite intensity						
Metabolite	M1	M2	M3	M4	M5		AJA^1^
							
(Molecular Ion)	415.5	413.5	415.5	397.5	575.5		399.5
							
Retention time (min)	8.0–8.2	8.4–8.5	9.7–10.2	12.9–13.1	13.9–14.4		13.6–14.4
							
Species	Incubation Time (min)						
Rat	0	ND	ND	ND	ND	ND	1.00
	120	ND	ND	ND	ND	ND	1.00
	240	ND	ND	ND	ND	ND	1.00
Monkey	0	ND	ND	ND	ND	ND	1.00
	120	trace	ND	ND	ND	0.006	1.00
	240	ND	ND	ND	trace	0.006	1.00
Dog	0	ND	ND	ND	ND	ND	1.00
	120	ND	ND	ND	ND	0.024	1.00
	240	ND	ND	ND	ND	0.024	1.00
Human	0	ND	ND	ND	ND	ND	1.00
	120	0.012	0.015	0.007	trace	ND	1.00
	240	0.014	0.017	0.009	0.001	0.001	1.00

ND, not detected.

1 AJA set at 1.00.

An extracted ion chromatogram of the incubation of AJA with human hepatocytes at 240 min is shown in Figure 2. Five metabolites with retention times at 8.00–8.20 min (M1), 8.40–8.50 min (M2), 9.70–10.20 min (M3), 12.90–13.10 min (M4), and 13.90–14.40 min (M5) were observed in the extracted ion chromatogram. M4 and M5 were only observed in the 240 min samples, but not in the 120 min samples; M1, M2, and M3 were seen at both 120 and 240 min.

Extracted ion chromatograms of incubation of AJA with canine hepatocytes at 0, 120, and 240 min are shown in Table 2. Extracted ion chromatograms of incubation of AJA with monkey hepatocytes at 0, 120, and 240 min are also shown in Figures 1 and 2, and Table 2. One metabolite (M5) was observed in the extracted ion chromatogram of both canine and monkey hepatocyte incubations in the 120 and 240 min samples. Trace M1 in the 120 min samples and trace M4 in the 240 min samples were detected in ion chromatograms from monkey incubations. Extracted ion chromatograms of incubations of AJA with rat hepatocytes at different times are shown in Table 2 where no metabolites were observed. Cell-free control incubation samples were analyzed by HPLC/MS (data not shown). No other peak except parent (AJA) was observed, indicating that AJA was stable in the cell-free incubation solution.

### Metabolite at 8.00–8.20 min (M1)

The deprotonated molecular ion of metabolite M1 at *m/z* 415.5 was 16 amu more than that of AJA (*m/z* 399.5) indicating that it was a hydroxylation product of AJA. The product ion spectrum of *m/z* 415.5 (MS^2^) showed a base peak at *m/z* 371 and a major peak at *m/z* 397, which was 16 amu greater than the major fragments of AJA (*m/z* 355, 381). The product ion spectrum of *m/z* 371 (MS^3^) showed a base peak at *m/z* 317 and a major peak at *m/z* 251 both of which were also 16 amu more than the major fragments of *m/z* 355 (*m/z* 301, 235). MS^2^ and MS^3^ data of M1 suggested that the hydroxylation occurred on the side chain of AJA, but not at the end carbon methyl group.

### Metabolite at 8.40–8.50 min (M2)

The deprotonated molecular ion of metabolite M2 was *m/z* 413.5; 14 amu more than that of AJA (*m/z* 399.5), which suggested that it was an oxidation product of AJA containing a keto group. The product ion spectrum of *m/z* 413.5 (MS^2^) showed a base peak at *m/z* 369 and a major peak at *m/z* 395, which were 14 amu more than the major fragments of CPL7075 (*m/z* 355, 381), but 2 amu lesser than that of M1. The product ion spectrum of *m/z* 369 (MS^3^) showed a base peak at *m/z* 315 and a major peak at *m/z* 249, which were also 14 amu more than the major fragments of *m/z* 355 (*m/z* 301, 235), but 2 amu lesser than that of *m/z* 371 (M1). MS^2^ and MS^3^ data of M2 suggested that it was the oxidation product of M1, and the oxidation occurred on the side chain of AJA. The proposed structures of those metabolites are shown in Figure 1.

### Metabolite at 9.70–10.20 min (M3)

The deprotonated molecular ion of metabolite M3 at *m/z* 415.5 was 16 amu more than that of AJA (*m/z* 399.5), and was the same as that of that of M1. The product ion spectrum of *m/z* 415.5 (MS^2^) showed a base peak at *m/z* 385 and major fragments of *m/z* 341, 371, and 397. The product ion spectrum of *m/z* 385 (MS^3^) showed a base peak at *m/z* 341, and a major peak at *m/z* 367. The product ion spectrum of *m/z* 341 (MS^4^) showed a base peak at *m/z* 287, and major peaks at *m/z* 179, 221, and 323. By comparing the MS^2^, MS^3^ data with those of both AJA and M1, metabolite M3 was assigned as another hydroxylation product of AJA, but the hydroxylation occurred on the end carbon methyl group of the side chain.

### Metabolite at 12.90–13.10 min (M4)

The deprotonated molecular ion of metabolite M4 at *m/z* 397.5 was 2 amu lesser than that of AJA (*m/z* 399.5), indicating that it was a dehydrogenation product of AJA, and may be formed from a hydroxylation product by loss of water. The product ion spectrum of *m/z* 397.5 (MS^2^) showed a base peak at *m/z* 353 and a major peak at *m/z* 379; both fragments were also 2 amu lesser than those of AJA. The product ion spectrum of *m/z* 353 (MS^3^) showed a base peak at *m/z* 299 and a major peak at *m/z* 233 and both fragments were also 2 amu lesser than those of 355, a major fragment of AJA. MS^2^ and MS^3^ data suggested that the dehydrogenation occurred on the side chain of AJA. However, the position of the dehydrogenation could not be determined based on the MS^2^, MS^3^ data.

### Metabolite at 13.90–14.40 min (M5)

The deprotonated molecular ion of metabolite M5 at *m/z* 575.5 was 176 amu more than that of AJA (*m/z* 399.5), suggesting that it was a glucuronide conjugate of AJA. The product ion spectrum of *m/z* 575.5 (MS^2^) showed a base peak at *m/z* 399, which was formed by a loss of mass 176 glucuronide. The product ion spectrum of *m/z* 399 (MS^3^) showed a base peak at *m/z* 355 and a major peak at *m/z* 381 exactly the same as those of AJA, indicating that a direct glucuronidation on either carboxylic acid or phenolic hydroxylß group of AJA had occurred.

The proposed metabolic pathways of AJA in canine, monkey, human hepatocytes are summarized as follows: glucuronidation of AJA to form its glucuronide conjugate (M5) was a common pathway for canine, monkey, and human hepatocyte metabolism. Other metabolic pathways in human hepatocyte incubations included hydroxylation of AJA on the carbon of the alkyl chain to form metabolite M1, which was oxidized to form a keto metabolite M2; hydroxylation of AJA on the end carbon methyl group of the alkyl chain to form metabolite M3; and dehydrogenation of AJA on the alkyl chain to form metabolite M4. Trace amounts of metabolites M1 and M4 were observed in monkey hepatocyte incubations. The proposed metabolic pathways of AJA in canine, monkey, and human hepatocytes are shown in Figure 1.

### Evaluation of the inhibitory potential of AJA against five principal human cytochrome P450 isozymes using human hepatic microsomes

The inhibitory potential of AJA on the activity of five principal human hepatic cytochrome P450 isozymes (CYP1A2, CYP2C9, CYP2C19, CYP2D6, and CYP3A4/5) was evaluated in pooled human hepatic microsomes at concentrations up to 50 μmol/L using phenacetin, tolbutamide, S-mephenytoin, dextromethorphan, and midazolam, and testosterone (both used to test CYP3A4/5), respectively, as the probe substrates (Table 2). Formation of the metabolites reflects CYP450 activity in the human hepatic microsomes. LC/MS/MS was used to quantify the metabolites of the probe substrates (acetaminophen for CYP1A2, 4-hydroxytolbutamide for CYP2C9, 4′-hydroxymephenytoin for CYP2C19, dextrorphan for CYP2D6, 1′-hydroxymidazolam, and 6ß-hydroxytestosterone for CYP3A4/5). AJA displayed no inhibition on CYP1A2, CYP2C9, CYP2C19, CYP2D6, and CYP3A4/5 activity with IC_50_ values greater than 50 μmol/L for each P450 isozyme. The mean IC_50_ values for the positive controls were 0.77, 0.24, 14, 76, 0.07, and 0.10 μmol/L (midazolam as substrate) and 0.06 (testosterone as substrate) for CYP1A2, CYP2C9, CYP2C19, CYP2D6, and CYP3A4/5, respectively. The IC_50_ values obtained in this study for the positive controls exhibited good agreement with reported literature values (Dierks et al. 2001).

**Figure 3:**
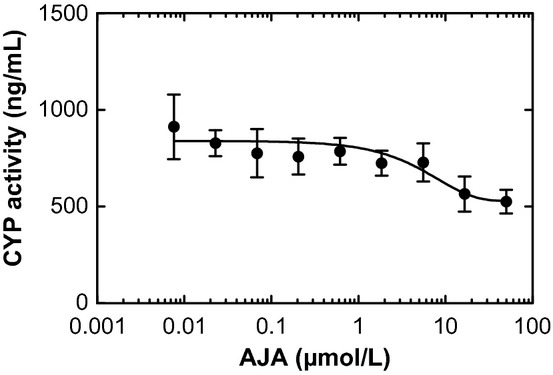
6ß-Hydroxytestosterone (CYP3A4/5 metabolite) concentrations in human hepatic microsomal incubations (substrate testosterone, 50 μmol/L). Values shown are the amounts of 6ß-Hydroxytestosterone measured using mass spectrometric analysis.

Table 3 summarizes the relative CYP450 activities in incubations treated with varying concentrations of AJA and positive controls. The results showed that AJA does not inhibit CYP1A2 or CYP2D6 activities at CPL7075 concentrations up to 50 μmol/L. There was a slight decrease in CYP2C9, CYP2C19, and CYP3A4/5 activities. The CYP3A4/5 activity, as represented by the testosterone metabolite, is shown in Figure 3. At 50 μmol/L of AJA, CYP activity as percent of vehicle control was 78% for CYP2C9, 76% for CYP2C19, and 76% and 55% for CYP3A4/5 using midazolam and testosterone, respectively, as substrates. The calculated IC_50_ values were >50 μmol/L for CYP1A2, CYP2C9, CYP2C19, CYP2D6, and CYP3A4/5. The positive controls were determined to be potent CYP450 inhibitors using selected probe substrates. The calculated IC_50_ values for positive controls were in good agreement with reported literature values (Dierks et al. 2001). AJA did not significantly inhibit CYP1A2, CYP2C9, CYP2C19, CYP2D6, and CYP3A4/5 activities since estimated or calculated IC-50 values were >50 μmol/L, the highest AJA concentration tested for all isozymes.

**Table 3:** Summary of the effects of AJA on human CYP isozyme activity

AJA inhibitory activity expressed as% vehicle control						
AJA (μmol/L)	CYP1A2	CYP2C9	CYP2C19	CYP2D6	CYP3A4/5^1^	CYP3A4/5^2^
50	114	78	76	111	76	55
16.6	114	95	89	115	69	59
5.55	96	92	80	102	79	76
1.85	85	102	87	96	81	76
0.617	85	98	84	99	86	82
0.206	92	94	85	83	82	79
0.069	85	88	81	82	83	81
0.0229	91	91	86	85	84	86
0.0076	97	94	103	98	84	95
0 (VC)	100	100	100	100	100	100
IC_50_ (μmol/L)	>50	>50	>50	>50	>50	>50

VC, vehicle control. CYP450 activity was defined as rate of metabolite formed (pmol/min/mg protein) at each AJA concentration, expressed as a percent of vehicle control (0 μmol/L). N = 3.

1 Midazolam was used as the probe substrate.

2 Testosterone was used as the probe substrate.

## Discussion and Conclusions

In summary, AJA was subjected to metabolite profiling analysis after in vitro incubation with rat, canine, monkey, and human hepatocytes. Unmetabolized AJA remaining after a 2-h incubation amounted to 103%, 90%, 86%, and 83% for rat, dog, monkey, and human hepatocytes, respectively. The incubation of AJA with human hepatocytes produced five metabolites, M1, M2, M3, M4, and M5 (Table 2 and Fig. 1) following 240 min of incubation. The incubation of AJA with rat hepatocytes did not generate any detectable metabolites. Only one metabolite of AJA, M5, was observed in the extracted ion chromatogram of canine hepatocyte incubations at 120 and 240 min. Metabolite M5 was observed in the extracted ion chromatogram of monkey hepatocyte incubations in both 120 and 240 min samples, a trace metabolite M1 was detected in the 120 min sample, and a second trace metabolite M4 was observed in the 240 min sample.

Metabolites M1 and M3 were identified as hydroxylation products of AJA; the hydroxylation positions were on the side chain, however, the exact positions could not be determined by MS/MS. The product ion spectra of M3 suggested that the hydroxylation was on the end carbon methyl group of the side chain. Similarly, an early study on the in vivo metabolism of THC in rabbits found side chain hydroxylation as one of the pathways (Burstein et al. 1972). M2 was characterized as an oxidation product of the M1 metabolite of AJA with a keto group on the side chain. M4 was identified as a dehydrogenation metabolite and the dehydrogenation was on the side chain. M5 was a glucuronide conjugate of AJA, and the glucuronidation occurred on either the carboxylic group or phenol group, or a mixture of both.

A report (Batista et al. 2005) using plasma samples obtained from a phase 2 trial of AJA (Karst et al. 2003) gave in vivo evidence for glucuronide formation as a metabolic pathway in humans following oral administration and representing at most 10% of the total drug present in the plasma at doses up to 40 mg b.i.d. Batista et al. (2005) did not report evidence for other possible routes of metabolism such as hydroxylation at various positions that is extensive among the cannabinoids in general. This is in agreement with the findings reported here showing minimal metabolism.

In a separate study, AJA showed low inhibitory activity against five principal human cytochrome P450 isozymes using human hepatic microsomes as the test system. The specific isozymes were CYP1A2, CYP2C9, CYP2C19, CYP2D6, and CYP3A4/5. These data suggest that AJA unlike other cannabinoids such as THC or cannabidiol (Yamaori et al. 2010, 2011, 2012) is not likely to interfere with the normal metabolism of drugs or endogenous substances thereby further supporting claims for its high safety profile.

## Abbreviations

AJA: ajulemic acid
ESI: electrospray ionization
HPLC: high-performance liquid chromatography
MS: mass spectrometry
MS^n^: tandem mass spectrometry
QPS: QPS, LLC
TIC: total ion chromatogram
%CV: coefficient of variation
CYP450: cytochrome P450
CYP1A2: cytochrome P450 isoform 1A2
CYP2C9: cytochrome P450 isoform 2C9
CYP2C19: cytochrome P450 isoform 2C19
CYP2D6: cytochrome P450 isoform 2D6
CYP3A4/5: cytochrome P450 isoform 3A4/5
IC_50_: inhibitor concentration which inhibits maximal activity by 50%
PAR: peak area ratio
VC: vehicle control (0 μmol/L test compound, 1% organic solvent)
%VC: percent of vehicle control = (Mean nmol/min/mg value at each Inhibitor Concentration)/(Mean nmol/min/mg value for Inhibitor Concentration at 0 μmol/L) × 100%

## Supporting Information

**Data S1.** The synthesis of ajulemic acid from dimethylheptylresorcinol (DMHR) and p-menthadienol (PMD).
