# Supplementary material for: In vitro metabolism and metabolic effects of ajulemic acid, a synthetic cannabinoid agonist
Source: Pharmacol Res Perspect. 2013 Dec 15;1(2):e00017. doi: 10.1002/prp2.17 (PMC4186433; doi:10.1002/prp2.17)
Supplement: Supplementary file 1 — Data S1. The synthesis of ajulemic acid from dimethylheptylresorcinol (DMHR) and p-menthadienol (PMD). [file prp20001-e00017-SD1.docx]

The Synthesis of Ajulemic acid from DIMETHYLHEPTYLRESORCINOL (DMHR) and P-MENTHADIENOL (PMD)

**Step 1**

To a 5-L, 3-neck round bottom flask was charged DMHR (6) (240g, 1 equivalents), *p-*toluenesulfonic acid (40.8g, 0.2 equivalents) and toluene (1400mL). To this was added PMD (170 g, 1.1 equivalents) over 1h, followed by a toluene (40mL) rinse while maintaining the batch temperature at 15–30°C. The batch was heated to 70–80°C under partial vacuum, and a Dean-Stark trap filled with toluene was used to remove water azeotropically while maintaining the same volume of toluene. The reaction was determined complete after 2 hours by HPLC, meeting the specification of ≤2.0% (AUC) cannabidiol. At atmospheric pressure, pyridine (13.4g, 1.6 equivalents) was added over ~15 min while maintaining batch temperature at 70–80°C. Acetic anhydride (165.9g, 1.6 equivalents) was then added to the reactor over ~20 min again maintaining the batch temperature at 70–80°C. After 2.5h the batch was sampled and passed the specification of ≤2.0% (AUC) of Compound 8. Room temperature water (1920mL, 8 equivalents) was added and the batch was held at ambient temperature overnight. At ~25°C, the phases were allowed to settle and the lower aqueous layer was removed. The batch was washed further with 480mL of room temperature water. A white emulsion was observed and a heat gun was required to produce a clean phase split. After the aqueous layer was removed, the reaction mixture was transferred to a 3-L, single-neck round bottom flask and placed on the rotary evaporator. The batch was concentrated down to ~600 g (~1/3 of original reaction mixture) and IPA (1.2L, 5 equivalents) was added. This was repeated 2 more times and then tested by NMR to determine the residual toluene level. The sample passed the specification of ≤2% toluene. The reaction mixture (1563g) was then split into several batches to do crystallization experiments. Half of the reaction mixture (782g) was crystallized from an 8:2 IPA/water solution (240mL IPA and 240mL water was added to obtain the ratio). The batch temperature was adjusted to 45–55°C. As it cooled down to 40–45°C, crystals were observed. The batch was further cooled down to 20–25°C at 10°C/h and held overnight. Then, the batch was placed in an ice bath, filtered and washed with pre-chilled IPA/water (400mL, 4:1 IPA/water). The cake was pulled dry on the filter and then transferred to the vacuum oven where it was dried at 45–55°C. The product was offloaded and gave Compound 9 (147.32g, 70.3% yield).

**Step 2**

To a 500-mL, 3-neck round bottom flask was charged Compound 2 (28.37g, 1 equivalents), Selenium dioxide (9.54g, 1.25 equivalents), tetrahydrofuran (122mL, 4.3 equivalents) and water (5.67mL, 0.2 equivalents). The reactor was heated to 55–65°C, and the reaction deemed complete at 23.5h by HPLC, passing the specification of ≤2.0% (AUC) Compound 2 by HPLC. The batch was cooled to 0–10°C at 10°C/h and then slowly treated with 35 wt% hydrogen peroxide (53.0 g, 3 equivalents) over 60 min while maintaining the batch temperature <25°C. The batch temperature was adjusted to 10–25°C and held at this temperature until the reaction met the specifications of ≤10.0% (AUC) of Compound 3 by HPLC. After a total time of ~18h the reaction was deemed complete and slowly quenched with 20 wt% sodium thiosulfate solution (108.73g, 2 equivalents) while maintaining the batch temperature <35°C. The batch was tested for presence of peroxide using Quantofix peroxide text strips and gave a negative result. The batch was then filtered through a pad of Celite. The reaction mixture stirred for 10min before the lower aqueous layer was removed. The batch was then washed with 10wt% sodium chloride (56.75mL, 2 equivalents) and the organic layer was transferred back into the cleaned 1-L reactor along with water (142mL, 5 equivalents). To this was added 50wt% sodium hydroxide solution (20.67g, 3.8 equivalents) while maintaining the batch temperature <55°C. The batch was heated to 45–55°C and held at this temperature for ~1h after which time it was sampled and met the specification of ≤2.0% (AUC) of Compound 4 by HPLC. Heptane (71mL, 2.5 equivalents) was added and the reaction mixture was allowed to cool to room temperature. Three phases were observed upon allowing the phases to settle. The bottom two layers were separated and charged with heptane (71mL, 2.5 equivalents). Again 3 phases were observed and the top organic layer was removed. The batch was held overnight. While maintaining the temperature ≤35°C, the pH of the aqueous mixture was adjusted to ~1 using 37 wt% hydrochloric acid (25.4g). Methyl *tert*-butyl ether (114mL, 4 equivalents) was added and the batch was stirred for 15min. The batch was allowed to settle for 5min, and the aqueous layer that settled out within 5min was removed. The remaining mixture was filtered through Celite and washed with ~30mL of MTBE. The layers were then separated and the organic layer was transferred to the rotary evaporator. The batch was concentrated down to ~62g and ~42mL (1.5 equivalents) of acetonitrile was added. This was repeated once more and the reaction mixture was concentrated down to the target mass of 62.05g, which is the mass of starting compound in 1.5 equivalents acetonitrile. The batch was cooled to ambient temperature and crystals were observed within 30 minutes. The batch was held overnight. (Note: Held overnight due to convenience.) It was then further cooled in an ice bath and held at 0– 5°C for ~4h. The batch was filtered and washed with pre-chilled acetonitrile (42mL, 1.5 equivalents) then dried under vacuum at 45–55°C. The product was offloaded and gave crude ajulemic acid (4.949g, 18.0% yield) with a purity of 96.4% (AUC) by HPLC (Method A).

**Step 3**

To a 50-mL reactor was charged crude ajulemic acid (3.45g, 1 equivalents) and heptane (20.7mL, 6 equivalents). To a 100-mL, 3-neck round bottom flask was charged pyridine (1.46mL, 2.1 equivalents) and heptane (6.9mL, 2 equivalents). Both reactors were then heated to 45–55°C. Over a period of 30min, the crude ajulemic acid dissolved in heptane was slowly transferred to the 100-mL reactor containing pyridine. Acetic anhydride (1.6g, 1.8 equivalents) was then added while maintaining the batch temperature at 45–55°C. The batch was stirred for ~2h then sampled to meet the specification of ≤0.5% (AUC) of crude ajulemic acid by HPLC. To the reactor was then slowly charged DI water (4.8mL, 1.4 equivalents) while maintaining the same batch temperature. The reaction mixture was stirred for 20h and sampled by HPLC to meet the specification of ≤0.5% (AUC) by HPLC. The reaction mixture was allowed to settle and the phases separated. Still at 45–55°C, the organic phase was charged with 1N HCl (9.7mL, 2.8 equivalents). After stirring for 30 min, the bottom aqueous layer was separated. The organic layer was washed with water (4.8mL, 1.4 equivalents) to obtain pH of ≥4. The batch was allowed to cool to 0–10°C at 10°C/h and held for ~3.5h. It was filtered, washed with pre-cooled heptane (20.7mL, 6 equivalents), and dried under vacuum at 45–60°C. The product was offloaded and gave Acetylated ajulemic acid (3.003g, 78.8% yield) with a purity of 97.8% (AUC) by HPLC (Method A).

**Step 4**

To a 250-mL, 4-neck round bottom flask was charged acetylated ajulemic acid (23g, 1 equivalents) and MTBE (92mL). 2N NaOH (62.5mL, 2.400 equivalents) was added while maintaining the batch temperature ≤55°C. The batch was maintained at 45–55°C and stirred for ~16h after which time the batch had no unreacted acetylated ajulemic acid and met the specification of ≤0.5% (AUC) by HPLC. The reaction mixture was allowed to cool to 25°C. While maintaining the batch temperature ≤25°C, the batch was acidified with 37wt% HCl until the pH was ≤1 (14.1g HCl). After stirring for 30min the lower aqueous layer was separated and the organic layer was washed with water (~25mL, ~1 equivalents). The pH of the water wash was ~4. The organic layer was then filtered through Celite and the cake was washed with MTBE (11.5mL, 0.5 equivalents). The reaction mixture was then heated to reflux to complete a solvent swap. After ~50mL of MTBE was distilled off, acetonitrile (100mL, 4.4 equivalents) was added. Again, ~50mL of solvent was removed and additional acetonitrile (100mL, 4.4 equivalents) was added. The solvent was then distilled down the pre-marked 4 vol. equivalents line. The solution was then allowed to cool to ambient temperature overnight before further cooling the batch to 0–10°C at 10°C /h. It was held for 4h, filtered, washed with pre-cooled acetonitrile (70mL, 3 equivalents), and dried in the vacuum oven under vacuum at 45–60°C with a slight nitrogen bleed. The product was assayed by GC and after 81h met the specification of ≤410ppm. The product was offloaded and gave ajulemic acid (19.592g, 91.8% yield) with a purity of 98.9% (AUC) by HPLC (Method A).

Figure 1. The system for metabolite profiling and identification consisted of a CTC LEAP HTC autosampler, a Surveyor HPLC pump, and an LTQ mass spectrometer. The HPLC/MS system was controlled by Xcalibur software. The following are the conditions for HPLC and mass spectrometry/HPLC.  Column type: Synergy 4u MAX-RP 80Å, 4.6 x 250 mm Gradient program: A: 0.1% NH4OH/high purity water B: 0.1% NH4OH/acetonitrile 1. 0 min: 75% A, 25% B 2. 15 min: 55% A, 45% B 3. 25 min: 10% A, 90% B 4. 30 min: 10% A, 90% B 5. 31 min: 75% A, 25% B 6.39min:75%A,25%B 800 lL/min. Ionization mode: Negative ESI


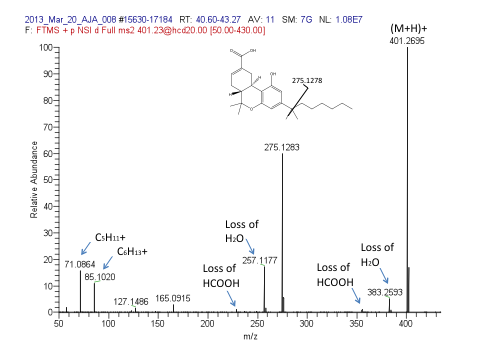


| Peak number | Relative retention time | AUC (%) |
| --- | --- | --- |
| 1 | 0.86 | 0.47 |
| 2 | 0.89 | 0.09 |
| 3 (AJA) | 1.00 | 98.9 |
| 4 | 1.12 | 0.15 |
| 5 | 1.14 | 0.12 |

**Table 1.** Purity of AJA. Product prepared by AMRI

using the procedure shown above.
